# Analyzing immune responses to varied mRNA and protein vaccine sequences

**DOI:** 10.1038/s41541-023-00684-0

**Published:** 2023-06-05

**Authors:** Hyeong-Jun Park, Yoo-Jin Bang, Sung Pil Kwon, Woori Kwak, Sang-In Park, Gahyun Roh, Seo-Hyeon Bae, Jae-Yong Kim, Hye Won Kwak, Yongkwan Kim, Soyeon Yoo, Daegeun Kim, Gyochang Keum, Eun-Kyoung Bang, So-Hee Hong, Jae-Hwan Nam

**Affiliations:** 1 Department of Medical and Biological Sciences, The Catholic University of Korea, Bucheon, Republic of Korea; 2 BK Plus Department of Biotechnology, The Catholic University of Korea, Bucheon, Republic of Korea; 3 SML Biopharm, Gwangmyeong, 14353 Republic of Korea; 4 Center for Brain Technology, Brain Science Institute, Korea Institute of Science and Technology, Seoul, 02792 Republic of Korea; 5 Department of Microbiology, College of Medicine, Ewha Womans University, Seoul, 07804 Republic of Korea

**Keywords:** Infectious diseases, RNA vaccines

## Abstract

In response to the COVID-19 pandemic, different types of vaccines, such as inactive, live-attenuated, messenger RNA (mRNA), and protein subunit, have been developed against SARS-CoV-2. This has unintentionally created a unique scenario where heterologous prime-boost vaccination against a single virus has been administered to a large human population. Here, we aimed to analyze whether the immunization order of vaccine types influences the efficacy of heterologous prime-boost vaccination, especially mRNA and protein-based vaccines. We developed a new mRNA vaccine encoding the hemagglutinin (HA) glycoprotein of the influenza virus using the 3′-UTR and 5′-UTR of muscle cells (mRNA-HA) and tested its efficacy by heterologous immunization with an HA protein vaccine (protein-HA). The results demonstrated higher IgG2a levels and hemagglutination inhibition titers in the mRNA-HA priming/protein-HA boosting (R-P) regimen than those induced by reverse immunization (protein-HA priming/mRNA-HA boosting, P-R). After the viral challenge, the R-P group showed lower virus loads and less inflammation in the lungs than the P-R group did. Transcriptome analysis revealed that the heterologous prime-boost groups had differentially activated immune response pathways, according to the order of immunization. In summary, our results demonstrate that the sequence of vaccination is critical to direct desired immune responses. This study demonstrates the potential of a heterologous vaccination strategy using mRNA and protein vaccine platforms against viral infection.

## Introduction

Owing to the rapid spread of SARS-CoV-2, the development of vaccines to reduce the morbidity and mortality associated with coronavirus disease (COVID-19) has been spurred in several countries. The RNA vaccine platform is a next-generation platform consisting of messenger RNA (mRNA) that encodes the target antigen. In 1990, Wolff et al. ^1^ first conceived the concept of an mRNA vaccine by demonstrating that protein can be sufficiently expressed in mouse skeletal muscle cells via direct injection of in vitro-transcribed mRNA. However, unstable and ineffective in vivo delivery combined with the high innate immunogenicity of mRNA, which hinders the translation of antigens, limited its application^2,3^. Nonetheless, technological advances in lipid nanoparticles (LNPs) and the introduction of nucleoside modification by pseudouridine have shown the potential to overcome these limitations^4,5^. mRNA does not integrate into the host genome, eliminating the need for insertional mutagenesis^6^; it can be manufactured in a cell-free manner, allowing rapid, scalable, and cost-effective production^7^.

Owing to these advantages, two COVID-19 mRNA vaccines (Moderna mRNA-1273 and Pfizer/BioNTech BNT162b2) were authorized by the US Food and Drug Administration (FDA) for emergency use in under a year. Therefore, these early licensed mRNA vaccines were given priority in many countries. Around the same time or later, various COVID-19 vaccines, including inactivated and viral vector vaccines, were approved, and globally administered. Furthermore, the FDA has recently approved a traditional protein subunit type vaccine, Novavax, for emergency use^8^. Because of the availability of these vaccines, people worldwide have inadvertently received a heterologous prime-boost vaccination schedule, i.e., the administration of different types of vaccines to achieve immunization against a single virus. It has been reported that heterologous prime-boost strategies induce more robust T-cell responses and higher neutralizing antibody titers^9^. Increasing clinical results indicate that heterologous vaccination strategies such as priming with viral vectored vaccines followed by boosting with mRNA vaccines or priming with inactivated vaccines followed by boosting with mRNA vaccines against COVID-19 provided enhanced immune responses compared to homologous vaccination strategies^10^. However, the immunogenicity and efficacy of heterologous priming-boosting using mRNA and protein vaccines have not yet been reported. It is also unknown how vaccine efficacy is affected by the sequence of immunization.

The influenza virus is one of the most important zoonotic viruses and has been estimated to cause ~3–5 million cases of severe illness and ~290,000–650,000 deaths every year globally^11^. Although current influenza vaccines are regarded as effective tools to protect against disease, their effectiveness will be greatly reduced if novel pandemic-causing viral strains emerge or we fail to predict the vaccine strain^12^. Therefore, an influenza mRNA vaccine that can be rapidly produced on a mass scale has been highlighted to be key in responding to influenza pandemics that might emerge in the future. Currently, a few monovalent (Sanofi MRT-5400, MRT-5401; Pfizer PF-07252220) or quadrivalent (Moderna mRNA-1010) influenza mRNA vaccines encoding hemagglutinin (HA) from seasonal influenza strains are undergoing clinical testing, and several others are in the preclinical phase^13^. Nevertheless, deducing an appropriate vaccination strategy along with increased available options is essential for preventing future pandemics.

In this study, our primary aim was to evaluate if the order of immunization with different vaccine types affects the efficacy of a heterologous prime-boost vaccination strategy. To achieve this, we developed a novel mRNA platform expressing the HA of the influenza virus using 3′-UTR and 5′-UTR of muscle cells (mRNA-HA). We tested its efficacy with a commercially available HA protein subunit vaccine (protein-HA) following homologous or heterologous immunization strategies to induce immune responses and protect from influenza infection. These results will provide insights into the rationale for the heterologous prime-boost strategy that was inevitably deployed during the pandemic.

## Results

### mRNA-HA priming and protein-HA boosting elicited balanced IgG1/IgG2a and high hemagglutination inhibition (HI) titers

**Fig. 1:**
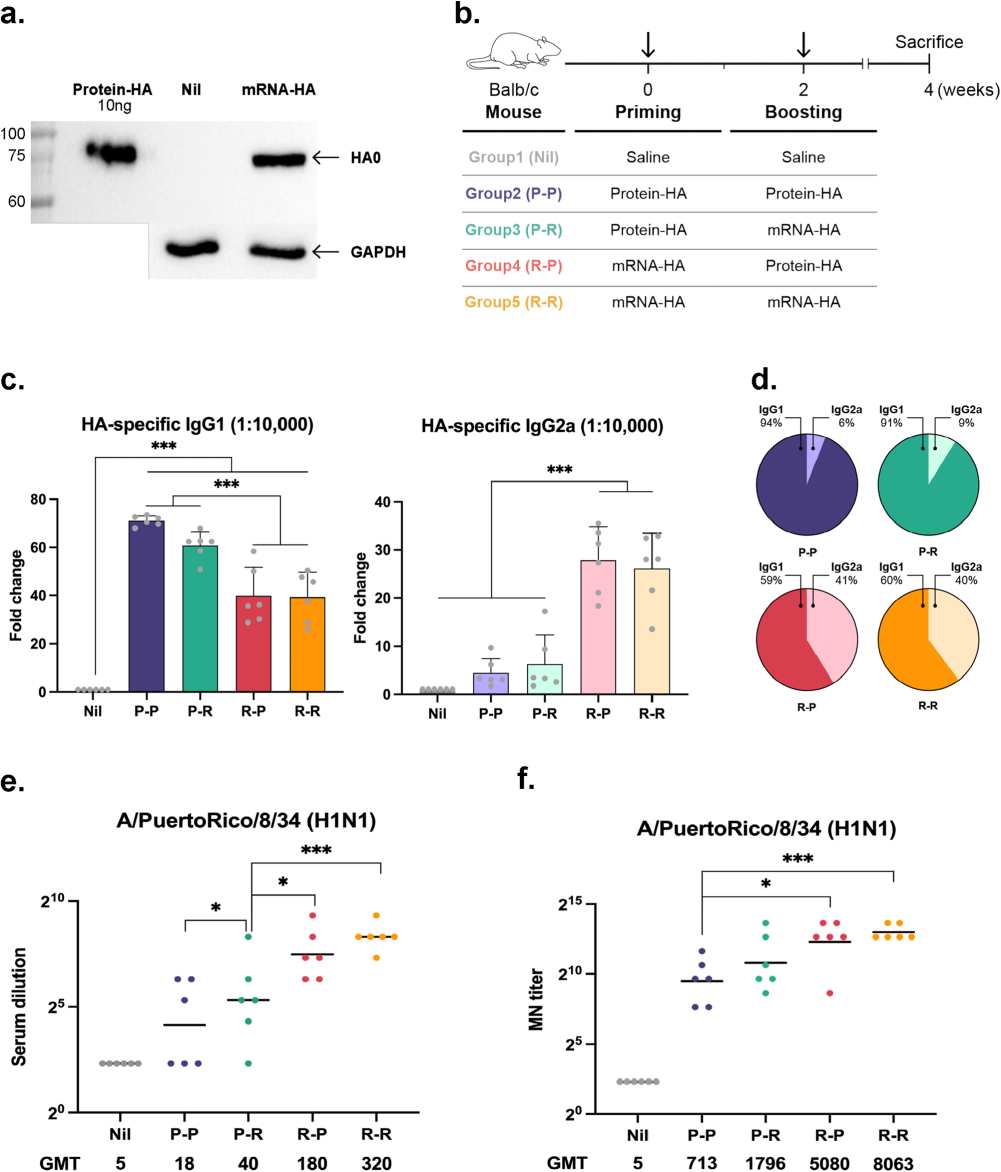
Evaluation of humoral immune responses induced by heterologous or homologous mRNA-HA or protein-HA immunization. **a** Hemagglutinin (HA) protein expressed in mRNA-HA transfected Vero cells. Recombinant HA protein was loaded as a positive control. **b** BALB/c mice were intramuscularly primed and boosted with LNP-formulated mRNA-HA (5 μg) or AddaVax-formulated HA protein (1 μg) at 2-week intervals, and then sacrificed 2 weeks after boosting. To measure IgG1, IgG2a levels, hemagglutination inhibition (HI), and microneutralization (MN) titers, sera were collected at indicated time points. **c** IgG1 and IgG2a levels measured using indirect ELISA. **d** The pie graph represents the ratio of IgG1 and IgG2a in mouse serum at sacrifice. **e** HI titer against vaccine strains measured by HI assay 2 weeks after the boost schedule. **f** MN titer against A/Puerto Rico/8/1934 measured by MN assay 2 weeks after the boost schedule; *n* = 6 mice; Data are represented as the mean ± standard deviation (SD). Statistical significance was analyzed using one-way ANOVA and Mann–Whitney U test. The significance of the differences between groups is indicated on the bars; *P < 0.05, ***P < 0.005.

We designed an mRNA platform that encodes the HA sequence of influenza strain A/Puerto Rico/8/1934 H1N1. The expression of the HA protein in Vero cells was confirmed after transfection with mRNA-HA using western blotting (Fig. 1a). Next, we compared humoral responses induced by homologous or heterologous vaccination with those induced by mRNA-HA or protein-HA, following the strategy shown in Fig. 1b. The sera were diluted 50- to 819,200-fold to set an endpoint at which antibodies were no longer detected, and then IgG1 and IgG2a levels were measured at a serum dilution of 1:10,000 (Supplementary Fig. 1a). As shown in Fig. 1c, mRNA-HA priming induced high levels of IgG2a, whereas protein-HA priming induced an IgG1-biased response. Balanced IgG1/IgG2a responses were observed in the heterologous mRNA-HA/protein-HA-immunized (R-P) and homologous mRNA-HA-immunized groups (R-R) groups (Fig. 1d). Because inducing neutralizing antibodies is a requirement for successful vaccine development, we checked the HI and microneutralization (MN) titers in the serum of each group. The R-P group showed higher levels of HI and MN titers than the P-R group, which were comparable to those of the homologous R-R group (Fig. 1e, f). The protein-HA homologous immunized group (P-P) showed the lowest HI and MN titers (Fig. 1e, f).

### mRNA-HA priming and protein-HA boosting induced strong T-cell responses

**Fig. 2:**
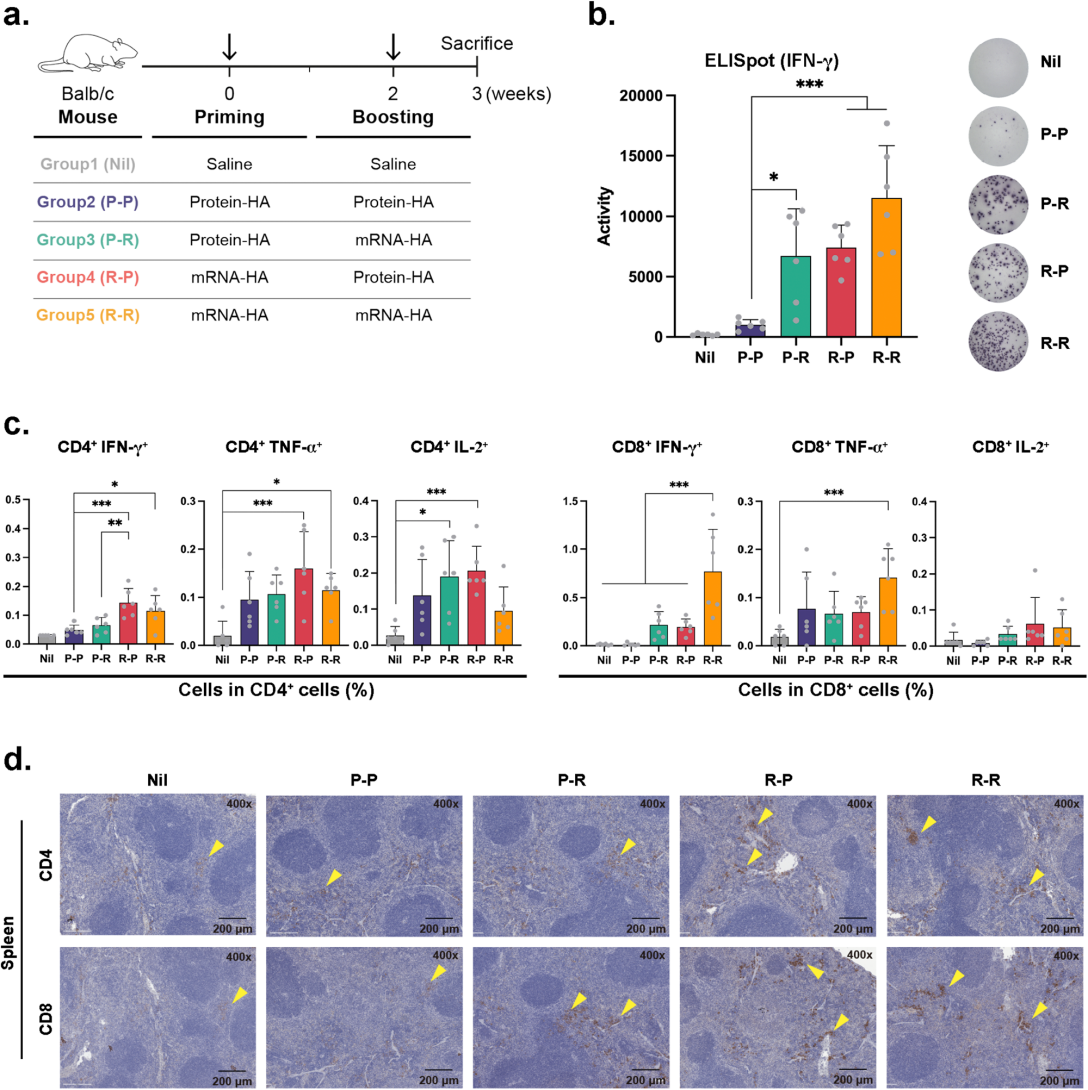
Analysis of T cells after the heterologous or homologous vaccination. **a** Immunization schedule of mice. BALB/c mice were intramuscularly primed and boosted with LNP-formulated mRNA-HA (5 μg) or AddaVax™ formulated HA protein (1 μg) at 2-week intervals and then sacrificed 1 week after boosting. **b** ELISpot assay shows IFN-γ-producing activity of splenocytes after stimulation with HA peptides. **c** Percentages of IFN-γ, TNF-α, and IL-2-producing CD4^+^ and CD8^+^ T cells in the spleen assessed using flow cytometry. **d** Immunohistochemical images of mouse spleen. Arrows indicate CD4^+^ or CD8^+^ T cells. Data are represented as the mean ± SD. Statistical significance was analyzed using one-way ANOVA. The significance of the differences between groups is indicated on the bars; *P < 0.05, **P < 0.01, ***P < 0.005.

Many studies have demonstrated that T cells play a key role in protective immunity against influenza viruses^14,15^. Therefore, we examined the T-cell responses induced by homologous or heterologous immunization with mRNA-HA and protein-HA. Mice were intramuscularly primed and boosted with LNP-formulated mRNA-HA (5 μg) or AddaVax™-formulated HA protein (1 μg) at 2-week intervals and then sacrificed 1 week after boosting (Fig. 2a). In this study, we chose AddaVax™ as an adjuvant as it is a squalene-based oil-in-water nano-emulsion similar to MF59 used in the influenza vaccine for the elderly and known to induce balanced Th1 and Th2 responses^16,17^. No statistical difference was observed in the enzyme-linked immunospot (ELISpot) activity of interferon-γ (IFN-γ) cytokine-producing cells in splenocytes between the R-P and P-R groups according to the immunization sequence, whereas the activity was significantly stronger in these groups than that in the P-P group (Fig. 2b). A similar result was obtained using IFN-γ enzyme-linked immunosorbent assay (ELISA; Supplementary Fig. 1b).

The frequency of antigen-specific IFN-γ producing cells among CD4^+^ T cells was higher in the R-P group than those in the P-P and P-R groups (Fig. 2c). Although it did not reach statistical significance, the frequency of antigen-specific tumor necrosis factor-α (TNF-α)-producing cells among CD4^+^ T cells was increased in the R-P group compared to those in the P-P and P-R groups (Fig. 2c). The frequencies of IFN-γ or TNF-α-producing cells among CD8^+^ T cells were significantly increased only in the R-R group, and the frequency of interleukin-2 (IL-2)-producing cells among CD4^+^ T cells was increased in the P-R and R-P groups compared to that in the control group (Fig. 2c). In addition, more CD4^+^ and CD8^+^ T cells were detected in the spleen tissues of the P-R and R-P groups than those in the P-P group (Fig. 2d).

### Heterologous prime-boost regime enabled the timely activation of distinct immune response

**Fig. 3:**
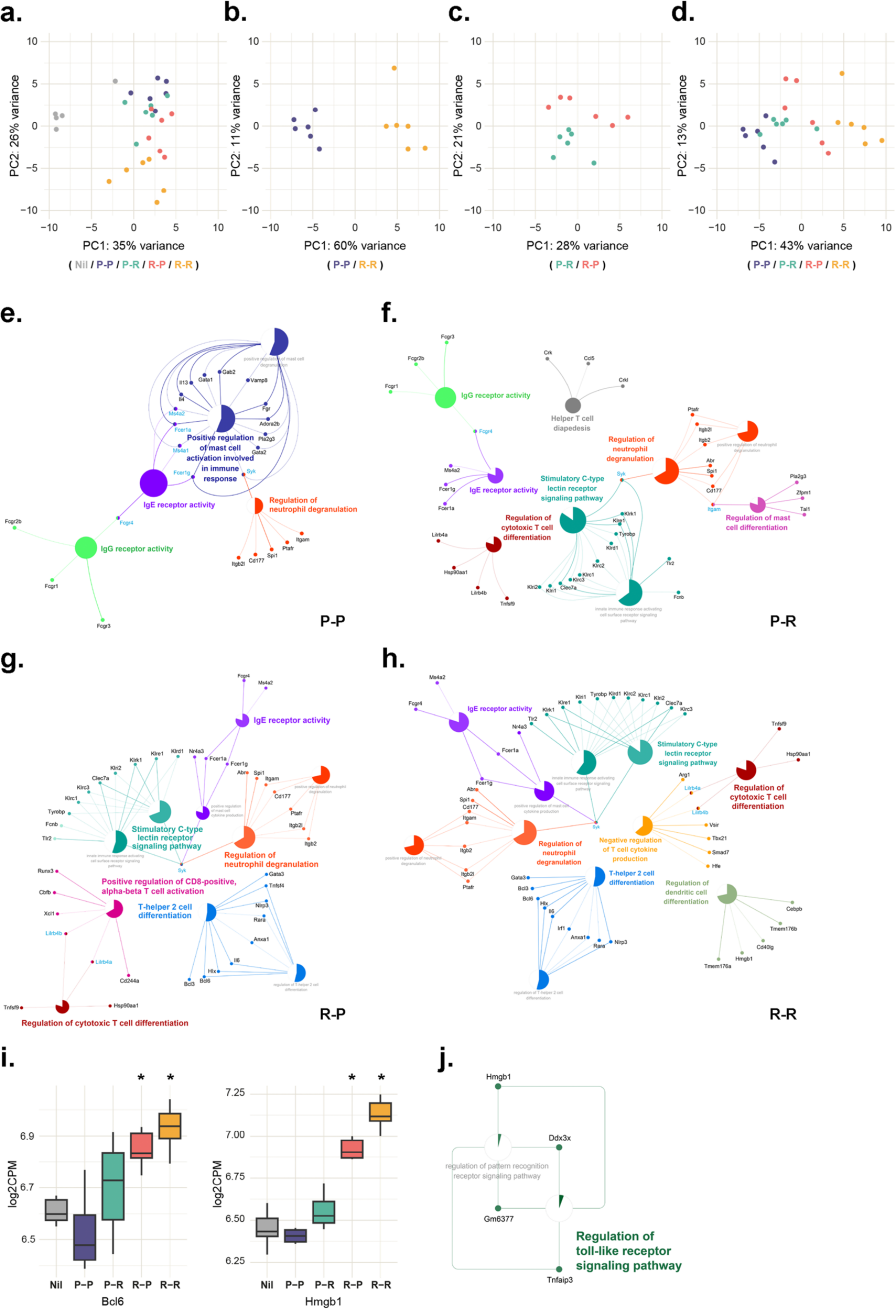
Transcriptome analysis for four different vaccination strategies 7 days after boosting. **a–d** Principal component analysis plot of data obtained for **a** all samples used in this study, **b** two homologous vaccination strategies, **c** two heterologous vaccination strategies, and **d** four groups with different vaccination strategies. **e–h** Network of enriched GO immune pathways and genes in **e** homologous protein-HA immunized (P-P) group, **f** protein-HA/mRNA-HA immunized (P-R) group, **g** mRNA-HA/protein-HA immunized (R-P) group, and **h** homologous mRNA-HA immunized (R-R) group. **i** Box plot for the expression level of two key DEG (*Bcl6, Hmgb1*) only significantly upregulated in the mRNA-primed group. **j** *Hmgb1-related* enriched GO immune pathway terms upregulated in the R-P group (mRNA-primed group) compared to the P-R group (protein-primed group). Data are represented as the mean ± SD. Statistical significance was analyzed using one-way ANOVA. The significance of the differences between groups is indicated on the bars; *P < 0.05.

To investigate the underlying mechanism of the heterologous prime-boost regime, we conducted RNA-seq of the splenic tissue samples of mice undergoing different vaccination regimes 7 days after boosting. In principal component analysis, different gene expression patterns were observed in the four vaccinated groups compared to the negative control (Fig. 3a). As shown in Fig. 3b and c, the gene expression patterns differed between the P-P and R-R groups but not between the P-R and R-P groups. Furthermore, the comparison of the gene expression patterns according to the prime-boost strategies revealed that the similarity of gene expression patterns depended on the type of vaccine used for priming (Fig. 3d). Gene Ontology pathway enrichment analysis using the Immune System Process database revealed increased mast cell and neutrophil degranulation pathways in the P-P group compared to that in the negative control group (Fig. 3e). The P-R group also showed an increase in mast cell and neutrophil degranulation pathways compared to the control group. Moreover, helper T-cell diapedesis, cytotoxic T-cell differentiation pathways, and stimulatory C-type lectin receptor signaling pathways were also increased in the P-R group than those in the control group (Fig. 3f). The R-P group showed enriched pathways similar to those in the P-R group; however, the regulation of Th2 differentiation and CD8^+^ T-cell activation pathways were increased in the R-P group compared to the P-R group (Fig. 3g). The R-R group showed increased innate immune response signaling, regulation of the dendritic cell pathway, and negative regulation of the T-cell cytokine production pathway, unlike other groups (Fig. 3h). Similar to the R-P group, the R-R group also showed increased regulation of the Th2 and cytotoxic T-cell differentiation pathways. Furthermore, the differentially expressed gene (DEG) analysis revealed that *Bcl6*, a gene encoding a transcription factor for follicular helper T cells, and *Hmgb1* were significantly increased in the mRNA-primed groups only compared to the negative control group (Fig. 3i). V-set immunoregulatory receptor and interferon regulatory factor 1 (*Irf1*) were specifically increased in the R-R group. Figure 3j shows the pathways and genes related to *Hmgb1* among the enriched pathways associated with genes that were significantly increased in the mRNA-primed group when the gene expression of the two heterologous immunized groups was compared.

### Evaluation of the protective efficacy of the heterologous priming-boosting regimen and analysis of immune responses after influenza infection

**Fig. 4:**
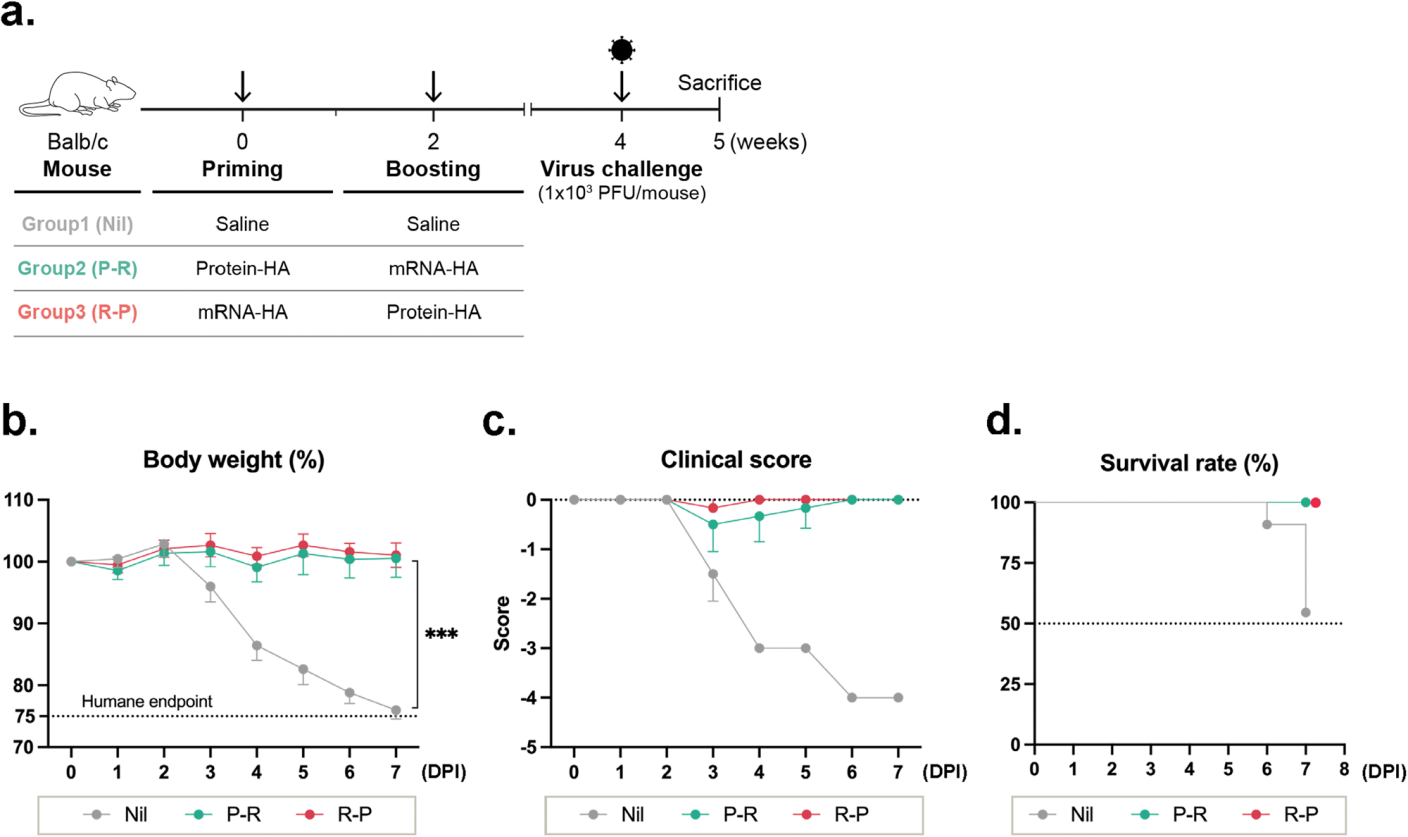
Heterologous prime-boost regimen comprising mRNA-HA and protein-HA vaccination sequence protects mice against the viral challenge. **a** BALB/c mice were challenged with influenza PR8 virus 2 weeks after the boosting and sacrificed after 1 week of the challenge. **b** Body weight loss of immunized mice, **c** clinical illness score, and **d** survival rate assessed 1 week after challenge with the influenza PR8 virus. Clinical illness score is detailed in “Methods” section. Data are represented as the mean ± SD. Statistical significance was analyzed using one-way ANOVA. The significance of the differences between groups is indicated on the bars; ***P < 0.005.

Next, we compared the protective efficacy of heterologous prime-boost regimes. Mice were subjected to priming and boosting as described previously (Fig. 1b) and challenged with PR8 virus 2 weeks after boosting (Fig. 4a). The prime-boost regimen with different vaccination sequences did not significantly affect body weight, clinical score, or survival rate (Fig. 4b–d). Although the induced HI titer in the P-R group was lower than that in the R-P group (Fig. 1e), both groups had mild symptoms, but no significant weight losses were observed. In hematoxylin and eosin (H&E)-stained spleen tissue, no difference in the size of the spleen or the ratio of white pulp was observed between the immunized groups. Vacuolation, indicative of spleen damage, occurred the least in the R-P group (Supplementary Fig. 2).

**Fig. 5:**
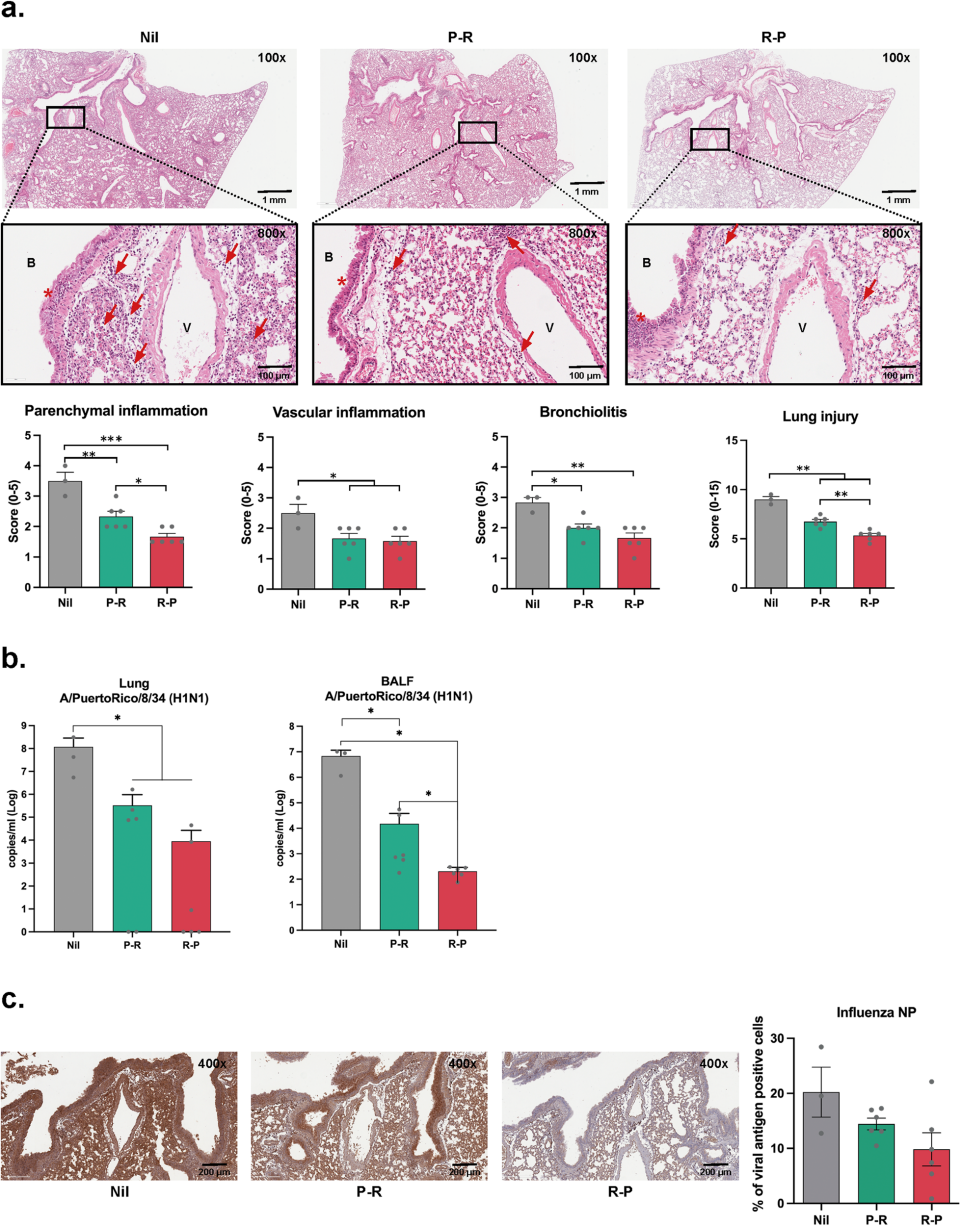
Histological analysis of lungs and spleens of mice immunized with heterologous prime-boost regimen after viral challenge. **a** Images of H&E-stained lung tissues and histological scores. B, bronchus, or bronchi; V, blood vessel; Arrow, inflammatory cells; Asterisk, epithelial mucus. **b** Viral loads in the lungs and bronchoalveolar lavage fluid (BALF) were measured using real-time PCR 1 week after the influenza PR8 challenge. **c** Immunohistochemical images of lungs stained with influenza A virus nucleoprotein (NP) specific antibody. Data are represented as the mean ± SD). Statistical significance was analyzed using one-way ANOVA. The significance of the differences between groups is indicated on the bars; *P < 0.05, **P < 0.01, ***P < 0.005.

Next, we assessed histopathological changes in all lung samples from the mice challenge study. One week post-challenge, all lung tissues from the virus-inoculated control group (Nil) showed severe histopathological changes characterized by bronchiolitis, inflammatory cell infiltration in the parenchyma, and epithelial hyperplasia (Fig. 5a). In contrast, mild to moderate (P-R) and minimal to mild (R-P) changes were observed in lung tissues collected from immunized mice. Moreover, viral loads in the lungs and bronchoalveolar lavage fluid (BALF) collected 1 week after the viral challenge were significantly reduced in the R-P group compared to that in the P-R group (Fig. 5b). Similar changes were observed in the lungs of mice stained with influenza A virus nucleoprotein (NP)-specific antibody (Fig. 5c). It seems that none of the vaccinated groups were perfectly protected against influenza infection, as viral N proteins and viral copies were detected in the lung and BALF. However, we believe that actual infectious virus titers are lower than the copy numbers measured by Real-time polymerase chain reaction (PCR), as it has been shown that viral copies are detected at high levels in Real-time PCR assays even when infectious viruses are not detected by median tissue culture infectious dose (TCID_50_) assay^18^. Thus, we expected that although 10–15% of cells were N protein-positive, some of them may have come from non-infectious virus particles. Furthermore, weight loss and clinical illness scores were not significant in either immunized group. Thus, we believe that both P-R and R-P immunization could induce potent protective immunity, and it seems that R-P induces better protective immune responses based on lung histology and viral copy number analyses.

**Fig. 6:**
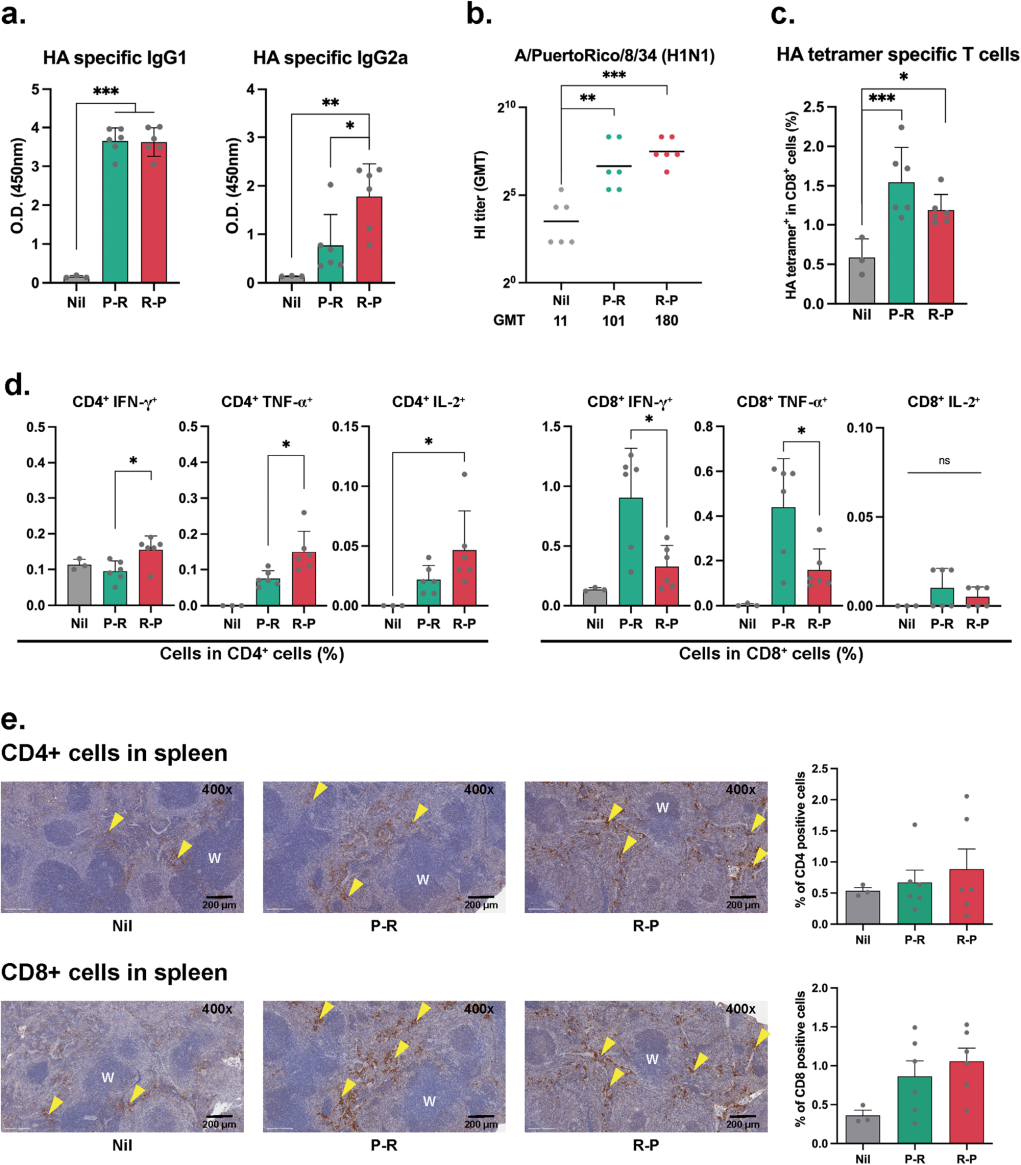
Analysis of humoral and T cell response after viral challenge. **a** IgG1 and IgG2a levels were measured by indirect ELISA using challenged mice sera. **b** HI titer against the influenza virus. **c** Percentages of HA tetramer-specific T-cells in the spleen of the challenged mice. **d** Percentages of IFN- γ, TNF-α, and IL-2 cytokine-producing CD4^+^ or CD8^+^ T cells. **e** Immunohistochemical images of spleen tissues of each group of mice. W, white pulp region; Arrowheads, CD4^+^ or CD8^+^ T cells. Data are represented as the mean ± SD. Statistical significance was analyzed using one-way ANOVA. The significance of the differences between groups is indicated on the bars; *P < 0.05, **P < 0.01, ***P < 0.005.

To further analyze the vaccine effectiveness after the viral challenge, we assessed the HA-specific IgG1, IgG2a, and IgA levels. The IgG1 level was similar, but the IgG2a level was higher in the R-P group than that in the P-R group. (Fig. 6a). No significant difference was observed between the two groups with respect to serum HA-specific IgA levels (Supplementary Fig. 3a). The HI titer tended to increase in the R-P group compared to that in the P-R group (*P* > 0.05; Fig. 6b). HA-specific CD8^+^ T cells were significantly increased in both P-R and R-P groups compared to those in the control group. However, no statistical difference was observed in the frequency of HA tetramer-specific CD8^+^ T-cells between the heterologous immune groups according to the immunization sequence (Fig. 6c). Interestingly, different patterns of CD4^+^ and CD8^+^ T-cell responses were observed between the two groups (Fig. 6d). The percentages of IFN-γ-, TNF-α-, and IL-2-producing CD4^+^T cells were higher in the R-P group than those in the P-R group. In contrast, the percentages of IFN-γ-, TNF-α-, and IL-2-producing CD8^+^ T cells were higher in the P-R group than those in the R-P group (Fig. 6d, e). ELISpot activity of IFN-γ cytokine-producing cells in the splenocytes of the heterologous prime-boosted groups was higher than that in the control group (Supplementary Fig. 3b). In addition, the frequencies of proliferating effector CD4^+^, CD8^+^, and central CD8^+^ T cells in the lungs after the viral challenge were the lowest in the R-P group compared to those in the control and P-R groups. This result indicated that the R-P group protected the lungs from virus infection (Supplementary Fig. 4).

## Discussion

Several studies have demonstrated that a heterologous prime-boost vaccination strategy is more effective than a homologous prime-boost strategy^10,19^. For instance, a heterologous vaccination strategy with COVID-19 mRNA and viral vector vaccines has been shown to induce higher levels of spike-specific neutralizing antibodies and T cells than a homologous vaccination strategy with only viral vector vaccines^9^. A recent study has demonstrated that a booster shot of the COVID-19 mRNA vaccine after two doses of inactivated vaccine significantly increased immune responses to SARS-CoV-2 and has been speculated to provide better protection against severe COVID-19 than three doses of inactivated vaccine^20^. However, no reliable data have been documented on the efficacy and rationale of heterologous prime-boost strategies using mRNA and protein platforms.

In this study, we developed an mRNA platform expressing the influenza HA protein and analyzed the immune responses induced by heterologous or homologous immunization strategies. Furthermore, we demonstrated that mRNA-HA priming and protein-HA boosting induced higher levels of IgG2a compared to protein-HA priming with mRNA-HA boosting. Our results supported the speculation that the sequence of immunization critically affects vaccine-induced humoral responses. We showed that protein-HA priming and mRNA-HA boosting (P-R) failed to increase IgG2a compared to homologous protein-HA immunization (P-P). In line with this, the HI titers in the P-R groups were lower than those in the R-P groups. Furthermore, vaccine-induced T-cell responses were differentially induced, depending on the immunization sequence. Antigen-specific IFN-γ and TNF-α-producing CD4^+^ T cells were increased in the R-P group 7 days after boosting. We also detected a significant increase in antigen-specific IFN-γ- and TNF-α-producing CD8^+^ T cells in the R-R group. These results suggest that homologous immunization with mRNA vaccines induces stronger CD8^+^ T-cell responses than heterologous prime-boosting with protein and mRNA vaccines. Although we did not conduct any experiment immunizing groups once with protein followed by two doses of mRNA, we expect that administering an additional two doses of mRNA after protein priming may resolve the imbalance between IgG1 and IgG2a and improve CD8^+^ effector responses, given that the highest T-cell responses and IgG2a levels were observed in the R-R group. Interestingly, after the challenge with the PR8 virus, a dramatic increase in IFN-γ- and TNF-α producing CD8^+^ T cells was detected in the P-R group, and a subdued increase was also detected in the R-P group. Consistent with previous studies, this study suggests that the mRNA vaccine boosting induces a CD8^+^ T-cell response^21,22^. However, unlike CD8^+^ T cells, cytokine-producing CD4^+^ T cells dramatically increased in the R-P group. Therefore, CD4^+^ or CD8^+^ biased T-cell responses could be induced depending on the order of immunization. Thus, it is necessary to determine whether to induce CD4^+^ or CD8^+^ T-cell responses depending on the features of the relevant infectious disease and establish optimal immunization order based on the immune responses that provide the best protection.

In addition, although no difference in clinical symptoms such as body weight, clinical score, or survival rate was observed 1 week after the challenge with the PR8 virus, the viral loads in the R-P group were lower than those in the P-R group. Although we did not check the titers of infectious virus in lungs and BALF, we believe that the pattern of infectious viral titers would be similar to that of viral copies and histological scores because histological scores of lungs and viral copy numbers have been shown to be correlated with infectious virus titer in many studies^18,23,24^. This result suggests that priming with an mRNA vaccine showed a more protective effect against influenza virus infection than priming with a protein vaccine. Interestingly, almost no anti-HA antibodies were detected in the naïve group after the viral challenge, which is consistent with our previous findings^25^. In both humans and mice, it takes more than two weeks after viral infection to produce sufficient antibodies. However, in this experiment, the mice were sacrificed and analyzed one week after the viral challenge, which may explain the lack of antibody response observed in the naïve group. Taken together, the present study demonstrated the characteristics of immune responses, and that the efficacy of vaccines can vary depending on the types of vaccines and immunization sequence. So far, numerous clinical studies have analyzed the immunogenicity of homologous or heterologous prime-boosting with COVID-19 vaccines. In general, the heterologous prime-boosting strategy with mRNA vaccine, viral vector vaccine, or inactivated COVID-19 vaccine induced more potent immune responses than homologous immunization in humans^9,19,26^. However, none of these studies have compared the immunogenicity dependent on the sequential order of administration of mRNA and protein COVID-19 vaccines. In our study, using a mouse influenza model, we found that using a heterologous prime-boost approach with protein and mRNA vaccines induced better T-cell responses and balanced humoral responses compared to homologous vaccination with protein. Similar to the results obtained by Chiu et al. ^26^, boosting with mRNA vaccine after protein priming induced higher levels of neutralizing antibodies than those induced by two doses of protein immunization. When compared to an mRNA homologous vaccination, mRNA priming-protein boosting also induced similar levels of neutralizing antibodies and IFN-γ- and TNF-α-positive T cells. Thus, we expect that heterologous vaccination may not necessarily induce better immune responses than homologous vaccination, and it may vary depending on the type of vaccine and virus.

In addition, we used the BALB/c mouse strain, which is commonly used to study influenza viruses, and the influenza virus used in our study is A/Puerto Rico/8/1934 H1N1, which has been adapted to the mouse model. As vaccine-induced immune responses could be different depending on the mouse strain and virus type used for the challenge, further studies using different mouse strains, as well as different virus infection models, are needed to confirm our findings.

Studies have shown that mRNA vaccines exhibit more adverse reactions than traditional vaccines. In particular, when inoculation is repeated, mRNA vaccines might cause adverse reactions, such as myocarditis or hypersensitivity reactions, more frequently than traditional protein vaccines^27,28^. However, because of their amenability to rapid design and large-scale manufacturing, mRNA vaccines could serve as the first line of defense to protect against the emergence of a new pandemic. Nevertheless, expanding the available vaccine options is required to reduce the incidence and mitigate the impact of future pandemics. Non-live vaccines, such as protein subunit vaccines, have a good safety profile compared to other vaccines, even in infants, the elderly, and pregnant women^29^. In addition, the low cost^30^ and stability of protein subunit vaccines under normal refrigeration conditions are advantageous^31,32^. Therefore, it is speculated that for continuous vaccination, if required with the emergence of new variants of coronavirus, protein vaccines may impart a safety advantage over mRNA vaccines. Owing to these benefits, several protein subunit type vaccines targeting the S or RBD proteins of SARS-CoV-2 are now under clinical trials; some, including Novavax, have obtained FDA approval for emergency use for people above 18 years of age^8^. Recently, a nanoparticle-based vaccine consisting of a two-protein component with self-assembling and RBD proteins was clinically approved for COVID-19 in Korea^33,34^. Therefore, the number of cases in which individuals were primed with Novavax’s protein-based vaccine and subsequently vaccinated with a COVID-19 mRNA vaccine, or vice versa, will increase over time. Analyzing these cases at a large scale will help us better understand the induction of immune responses by heterologous vaccination.

In conclusion, our findings suggest a heterologous vaccination strategy with the first inoculation of an mRNA vaccine to defend against the infectious disease, followed by a secondary or tertiary inoculation with a protein vaccine that requires time to be produced. This might be the best way to develop a safe and efficient vaccination strategy against the virus.

## Methods

### Mice

Six-week-old female BALB/c mice were obtained from Daehan Biolink Co. Ltd. (Seoul, South Korea). The mice were acclimatized for 1 week immediately after they were brought to the Catholic University of Korea before starting the experiment. Mice were housed under pathogen-free conditions with a 12/12 h light/dark cycle, a temperature of 23 °C ± 2 °C, and a relative humidity of 50% ± 10%. All animal experimental procedures in this study followed the guidelines of, and were approved by, the Institutional Animal Care and Use Committee of the Catholic University of Korea (CUK-IACUC-2022-020).

### Design and synthesis of mRNA-HA

The DNA template for the mRNA vaccine was a DNA fragment encoding the HA protein of the influenza A virus (A/Puerto Rico/8/1934). DNA templates of the mRNA vaccine were cloned into a plasmid vector with backbone sequence elements (T7 promoter, 5′- and 3′-UTR, 100 nucleotide poly(A) tail) interrupted by a linker (A50LA50, 20 nucleotides) to improve RNA stability and translational efficiency. The DNA was purified, spectrophotometrically quantified, and in vitro-transcribed with an EZ™ T7 High Yield In Vitro Transcription kit (Enzynomics, Daejeon, South Korea) and a Cap 1 capping analog (SMARTCAP®, ST PHARM, Seoul, South Korea) and with N1-methylpseudouridine-5′-triphosphate (m1ΨTP; TriLink, CA, USA) to replace uridine-5′-triphosphate (UTP).

After transcription, RNA was purified by lithium chloride precipitation. dsRNA was eliminated by cellulose-based purification^35^. RNA integrity was assessed using gel electrophoresis, and the concentration, pH, and endotoxin levels of the solution were determined.

### mRNA transfection and western blot

Vero cells were seeded at a density of 1 × 10^6^ cells/well in 6-well plates and incubated overnight. Afterward, 10 μg mRNA was transfected into each well using Lipofectamine2000™ (Invitrogen, MA, USA) according to the manufacturer’s instructions. HA protein (10 ng; Cat:11684-V08H; SinoBiological, Inc., Beijing, China) was used as a positive control and was detected using western blotting with the influenza A H1N1 hemagglutinin antibody (Cat:11684-R107; SinoBiological, Inc.). Primary antibodies were diluted as 1:1,000 in phosphate-buffered solution containing 0.1% Tween20 (0.1% PBST). The secondary antibodies were anti-rabbit antibody conjugated with horseradish peroxidase (HRP) (Cat: A120-101P, BETHYL, TX, USA) and diluted as 1:3,500 in 0.1% PBST. Unprocessed images of the western blots are provided in Supplementary Fig. 5.

### LNP formulation of the mRNA-HA

LNPs were prepared as per a reported protocol^36^. Briefly, all lipid components were dissolved in ethanol at a molar ratio of 25:25:10:38.5:1.5 (SM-102; 6,6′-trehalose dioleate; 1,2-dioleoyl-*sn*-glycero-3-phosphoethanolamin (DOPE); butyl lithocholate; and 1,2-dimyristoyl-*rac*-glycero-3-methoxypolyethylene glycol-2000 (DMG-PEG2000)), and mRNAs were dissolved at a charge ratio of N/P = 3 in sodium citrate buffer (50 mM; pH 4) solution^37^. LNPs were formulated using NanoAssemblr® Ignite^TM^ (Precision Nanosystems, BC, Canada) by mixing the aqueous and organic solutions at a ratio of 3:1 and a total flow rate of 10 mL/min. The solution of LNPs was concentrated by ultrafiltration using Amicon Ultra centrifugal Filter (UFC9030, Merck Millipore, MA, USA) following the manufacturer’s instructions.

### Characterization of LNPs

The size and zeta potential of the LNPs were determined using ZetaSizer Ultra (Malvern Panalytical, Malvern, UK). All the size and zeta potential data were obtained in triplicates (Supplementary Table 1). The mRNA encapsulation efficiency was analyzed using the Quant-iT RiboGreen RNA kit (Thermo Fisher Scientific, MA, USA). Briefly, mRNA-LNPs were lysed with 0.5% Triton-X or left untreated, followed by treatment with RiboGreen reagent following the manufacturer’s instructions. The quantity of mRNA in the samples was measured using a microplate reader (Spark®, TECAN, Mannedorf, Switzerland). The calculated encapsulation efficiency of mRNA was approximately 87%.

### Immunization

BALB/c mice were immunized intramuscularly in the upper thigh twice (prime and boost) at an interval of 2 weeks, with HA protein (1 µg) or mRNA-HA (5 µg). The HA protein used in our study was translated from a DNA sequence that encodes the hemagglutinin of Influenza A virus (A/Puerto Rico/8/1934 (H1N1)), specifically the sequence ABD77675.1, which includes Met1-Gln528. The protein was expressed in HEK293 cells and was formulated with AddaVax™ in a 1:1 ratio (v/v) by InvivoGen (CA, USA). mRNA-HA was formulated using LNP. The negative control group was injected with saline solution. For the T-cell analysis experiment, mice were immunized at 2-week intervals and sacrificed 1 week after boosting.

### ELISA

Antigen-specific antibody levels were measured using ELISA. Subsequently, HA protein (50 ng) was coated onto 96-well transparent plates. To identify the endpoint, sera were diluted 1:50 to 1:819,200, the secondary antibody was diluted 1:5,000, and total IgG was measured using antimouse IgG antibody (Cat: A90-103P, BETHYL). The dilution factor was set to 1:10,000, and antigen-specific IgG1 and IgG2a levels were measured using anti-mouse IgG1 and IgG2a antibodies conjugated with HRP, respectively (1:5,000 dilution, Cat: A90-105P, BETHYL or Cat: NB7516, NOVUS biologicals, CO, USA). IgA levels were measured using antimouse IgA antibody conjugated with HRP (1:5,000 dilution, Cat: A90-103P, BETHYL) in the sera at a 1:50 dilution.

### HI assay

The mouse sera were treated with a receptor-destroying enzyme (Denka Seiken, Tokyo, Japan) for 16 h at 37 °C and then inactivated for 30 min at 56 °C to eliminate non-specific responses. All sera were then serially diluted 2-fold with cold PBS in 96-well V-bottom plates (Corning, NY, USA) and incubated with standardized viral suspensions (4 HA U/25 μL) at 25 °C for 1 h. Chicken red blood cells (50 μL of 1%) were added, and the plates were incubated for at least 30 min at room temperature (24 °C). The geometric mean of the antibody titers (Geometric mean titers) was expressed as the reciprocal of the highest serum dilution that showed complete agglutination inhibition. When the initial dilution was 1/10, the lower limit of the detectable antibody titer was 1:10. Titers <1:10 were assigned a value of 1:5 for calculation.

### MN assay

The mouse sera were inactivated for 30 min at 56 °C. All sera were then serially diluted 2‐fold in serum-free medium starting from 1:10 in 96-well cell culture plates and incubated with an equal volume of viral suspension containing influenza H1N1 (100 TCID_50_) at 37 °C for 1 h in a humidified atmosphere with 5% CO_2_. After incubation, the mixture at each dilution (50 μL) was added to Madin-Darby Canine Kidney cell monolayer and incubated at 37 °C for 2 h in a humidified atmosphere with 5% CO_2_. Subsequently, RPMI-1640 medium (50 μL) containing 2% FBS was added, and the plates were incubated for 24 h at 37 °C in the same conditions. After incubation, the supernatant was carefully discarded, and 4% formaldehyde solution (100 μL; DeJong, Sheung, South Korea) was added. After a 4-h incubation at room temperature, the 4% formaldehyde solution was discarded, and 0.1% crystal violet solution (100 μL; Sigma-Aldrich, MO, USA) was added before incubation for 30 min. Afterward, the crystal violet solution was discarded, and the cell monolayer was washed with tap water. The results were evaluated under a microscope (Olympus, Tokyo, Japan), and the highest protective serum dilution ratio was considered as the neutralization titer.

### ELISpot assay

ELISpot was conducted using an ELISpot Plus mouse IFN-γ (ALP) kit (3321-4APW; Mabtech, OH, USA). Approximately 2.5 × 10^5^ splenocytes were seeded in 48-well cell culture plates and stimulated with 2 µg/well HA-specific T cell epitope peptide mixture (IYSTVASSL, LYEKVKSQL, DYEELREQL, SFERFEIFPKE, HNTNGVTAACSH, KLKNSYVNKKGK, NAYVSVVTSNYNRRF, and CPKYVRSAKLRM) for 24 h at 37 °C in a cell incubator. After 24 h, each step was performed following the manufacturer’s instructions. Briefly, after 24 h, a biotinylated IFN-γ detection antibody was used as the primary antibody and streptavidin-ALP antibody as the secondary antibody. Spot development was stopped when discrimination between groups was possible, and spots were counted with ELISpot 7.0 iSpot software (Autoimmun Diagnostika GmbH, Strassberg, Germany).

### Flow cytometry

For flow cytometry, 1 × 10^6^ splenocytes were seeded in a 96-well round-bottomed plate. Cells were stimulated in the same manner as they were in the ELISpot assay. One hour after peptide stimulation, GolgiPlug™ (BD, NJ, USA) was added to each well following the manufacturer’s instructions. The viability of the splenocytes was measured using the LIVE/DEAD™ Fixable Aqua Dead Cell Stain Kit (L34966, Invitrogen, MA, USA). After 5 h of peptide stimulation, the cells were blocked and stained using the following antibodies diluted at a ratio of 1:200 ~ 1:400: CD16/CD32 (Cat: 14-0161-82, Invitrogen), CD103-eFlour450-labeled (Cat: 48-1031-82, clone 2E7, Invitrogen), CD4-APC-Cy7-labeled (Cat: 25-0041-82, clone GK1.5, eBioscience, CA, USA), CD8-BV605-labeled (Cat: 100744, clone 53-6.7, BioLegend, CA, USA), IFN-γ-PE-Cy7-labeled (Cat: 505825, clone XMG1.2, BioLegend), TNF-α-FITC labeled (Cat: 506303, clone MP6-XT22, BioLegend), IL-2-APC-labeled (Cat: 503809, clone JES6-5H4, BioLegend), CD4-PE-Cy5-labeled (Cat: 100409, clone GK1.5, BioLegend), CD8a-FITC-labeled (Cat: 100705, clone 53.6.7, BioLegend), CD44-APC-Cy7-labeled (Cat: 103027, clone IM7, BioLegend), CD62L-PE-Cy7-labeled (Cat: 104417, clone MEL-14, Biolegend), and Ki-67-PE-labeled (Cat: 652403, clone 16A8, BioLegend).

To detect HA-specific T cells, the cells were blocked with streptavidin (Invitrogen) and CD16/32 for 20 min at 4 °C. After incubation, the cells were stained with H-2Kd HA tetramer-PE labeled with the HA533-541 IYSTVASSL (produced and generously provided by the National Institute of Health), CD8a-FITC-labeled, CD4-APC-Cy7-labeled, and CD45-PerCP-Cy5.5-labeled (Cat: 45-0451-82, clone 30-F11, eBioscience) antibodies and Fixable Viability Dye eFluor520 (Cat: 65-0867-18, eBioscience) for 30 min at 4 °C in the dark. All antibodies were treated with 0.1 µg for each sample. The cells and data were analyzed using a flow cytometer (FACS CytoFLEX, Beckman Coulter, CA, USA) and CytExpert (version 2.4, Beckman Coulter), respectively. Schematics of the gating strategy are provided in Supplementary Fig. 6.

### Transcriptome analysis

The spleen samples from each group, with six biological replicates, were obtained from immunized mice and stored in RNAlater™ stabilization solution (Invitrogen) for transcriptome analysis. Sequencing libraries for data generation were constructed using the Illumina TruSeq Stranded mRNA Library Prep Kit following the manufacturer’s protocol, and 101 bp paired-end sequencing was conducted using Illumina Novaseq6000. The library construction and data generation were performed at Macrogen (Seoul, South Korea). Sequencing artifacts and low-quality bases in the generated reads were removed using Trimmomatic^38^ with Truseq3-PE adapter sequences and LEADING:5 TRAILING:20 SLIDINGWINDOW:4:15 MINLEN:75 parameters. After filtering, the reads were mapped to the GRCm39 reference genome (https://www.ncbi.nlm.nih.gov/grc) using HISAT2^39^ with default parameters. The read counts for each gene were calculated using featureCounts^40^ in the Subread package. The plotPCA function in the DESeq2 package^41^ was used for principal component analysis, and differentially expressed genes (DEGs) were identified using EdgeR^42^. Each gene was tested for differential expression based on the generalized negative binomial model using the glmLRT function, and an adjusted *P* value < 0.01 (Benjamini-Hochberg False Discovery Rate, BH FDR) was applied for DEG identification. To identify the related functional pathways and networks of DEGs, the ClueGO app^43^ of Cytoscape^44^ was used for gene ontology (GO) pathway analysis^45^. Enriched terms were identified using a specific GO database—the Immune System Process database (2022-05-25)—with BH FDR < 0.01, and genes related to the enriched GO terms were identified using CluePedia^46^.

### Virus and viral challenge experiment

The A/H1N1 virus, A/Puerto Rico/8/1934, used for viral infection challenges, was generously provided by Dr. Seong BL of Yonsei University, Seoul, Korea. The heterologously immunized mice were challenged intranasally with 1 × 10^3^ plaque-forming units (PFU) of mouse-adapted A/Puerto Rico/8/1934 H1N1 virus in saline (50 µL) using a pipette. After the challenge, the body weight, survival, and clinical illness of mice were assessed. Clinical illness was scored using the following scale: 0 = no visible signs of disease; –1 = slight ruffling of fur; –2 = ruffled fur, reduced mobility; –3 = ruffled fur, reduced mobility, and rapid breathing; and –4 = ruffled fur, minimal mobility, huddled appearance, and rapid and/or labored breathing. Animals were sacrificed when their body weight decreased by more than 25% of their original body weight.

### Real-time PCR for determining viral loads

Mouse lungs were lavaged using a 22-gauge catheter and 1 mL saline by flushing the airway compartment three times. The BALF was centrifuged at 20,000× *g* for 10 min at 4 °C. Total RNA from the lungs and BALF was extracted using TRIzol^®^ reagent (Favorgen, Ping-Tung, Taiwan). The PCR reaction mix (25 μL) comprised 12.5 μL 2X SuperScript III Platinum Master Mix (Invitrogen), 2 μL of the mixture comprising forward primer (10 μM), reverse primer (10 μM), and dual-labeled probe (5 pmol), 0.5 μL SuperScript III Taq polymerase (Invitrogen), and 10 μL template RNA, standard, or negative control. Real-time PCR was performed on a Bio-Rad thermocycler CFX96 (Bio-Rad Laboratories Inc., CA, USA). The PCR conditions were as follows: 30 min at 50 °C and 5 min at 95 °C, followed by 45 cycles of 20 s at 95 °C, and 1 min at 55 °C. For virus detection, we used two pairs of influenza virus-specific primers (forward 5′-GACCRATCCTGTCACCTCTGAC-3′, reverse 5′-AGGGACTTYTGGACAAAKCGTCTA-3′) and TaqMan probes (5′-FAM-TGCAGTCCTCGCTCACTGGGCACG-BHQ1) designed based on the conserved matrix gene region of influenza A virus.

### Histopathological analysis

Sectioned lungs and spleens from experimental mice were submerged in 10% neutral buffered formalin, dehydrated, paraffin-embedded, and sliced into 4-μm-thick sections for histopathological examination. Histological images were obtained and evaluated using the Aperio ImageScope version 12.4 (Leica Biosystems Pathology Imaging, Buffalo Grove, IL, USA). The score of parenchymal inflammation quantifies inflammatory cell infiltration and loss of airspace (emphysema); the score of vascular inflammation indicates inflammatory cell infiltration and vessel edema; and bronchiolitis includes inflammatory cell infiltration, epithelial hyperplasia, congestion of bronco-mucosa, and focal erosion in the lung. In the spleen, the score of vacuolation reflects necrosis by macrophages, and it is accompanied with pigment deposition. The severity of histological changes was determined using a 5-point scoring system^47^ as follows: 0, no abnormality detected (NAD); 1 = minimal; 2 = mild; 3 = moderate; 4 = moderately severe; and 5, severe. The distribution was recorded as focal, multifocal, and diffused. Recruitment of inflammatory cells and morphological alterations in the lungs and spleen were assessed after H&E staining under a light microscope.

### Immunohistochemistry

The prepared lung and spleen sections were deparaffinized using xylene, and citrate buffer antigen (epitope) retrieval was performed following the procedures described previously^48^. Subsequently, a hot plate with a staining dish containing citrate buffer (10 mM; pH 6.0) was pre-heated to 95–100 °C. Slides were immersed in the staining dish, and the lid was loosely placed on the staining dish and incubated for 20 min. The staining dish was then removed from the hot plate (by turning it off) and incubated at room temperature for 20 min to cool the slides. Afterward, the slides were incubated in methanol and 3% H_2_O_2_ at room temperature for 30 min to block endogenous peroxidase activity. Non-specific binding of immunoglobulin was blocked by incubating the slides with 2.5% normal horse serum blocking solution (Cat: S-2012-50, Vector Laboratories, Inc., CA, USA) at room temperature for 1 h in a humidity chamber. Afterward, the slides were incubated with primary antibodies—CD4 monoclonal antibody (1:100 dilution, Cat: 14-0041-82, GK1.5; Invitrogen), CD8a monoclonal antibody (1:100 dilution, Cat: 14-0081-82, 53-6.7, Invitrogen), and Influenza A NP polyclonal antibody (1:200 dilution, Cat: PA5-32242, Invitrogen)—overnight at 4 °C. This was followed by incubation with biotinylated universal secondary antibody (1:50 dilution) and ABC reagents (1:50 dilution; Cat: PK-6100, Vector Laboratories, Inc.) for 1 h at room temperature in a humidity chamber. Finally, DAB (3,3’-diaminobenzidine) staining was performed by incubating the slides with the DAB working solution (DAB Peroxidase Substrate kit; Cat: SK-4100, Vector Laboratories, Inc.) for 3–10 min at room temperature. All sections were rinsed in 0.05% PBS more than three times between the steps.

The IHC images were obtained using Aperio ImageScope version 12.4, while the immunoreactive cells were calculated using Image J (Version 1.53t, NIH, http://rsb.info.nih.gov/ij/index.html). To analyze the percentage of antigen-positive cells, IHC slide scanner images were exported, and the brown image was converted into a black-and-white binary mask image. In order to accurately count the antigen-positive cells, the minimum size used to analyze particles was 5 pixels.

### Statistical analysis

One-way ANOVA with the Bonferroni *post hoc* test was performed for multiple-group comparisons, and the Mann–Whitney *U* test was used to compare the two groups. Differences were considered statistically significant at *P < 0.05, **P < 0.01, ***P < 0.005. Data are expressed as the mean ± standard deviation (SD). All statistical analyses were performed using GraphPad Prism 9 (GraphPad Software Inc., CA, USA).

### Reporting summary

Further information on research design is available in the Nature Research Reporting Summary linked to this article.

## Supplementary information

Supplementary information

REPORTING SUMMARY

## Data Availability

Transcriptome data that support the findings of this study have been deposited in the Sequence Read Archive with the accession number PRJNA973236. The data that support the findings of this study are available from the corresponding author upon reasonable request.
