# Supplementary material for: Analyzing immune responses to varied mRNA and protein vaccine sequences
Source: NPJ Vaccines. 2023 Jun 5;8:84. doi: 10.1038/s41541-023-00684-0 (PMC10239716; doi:10.1038/s41541-023-00684-0)
Supplement: Supplementary file 1 — Supplementary information [file 41541_2023_684_MOESM1_ESM.pdf]

SUPPLEMENTARY FIGURES

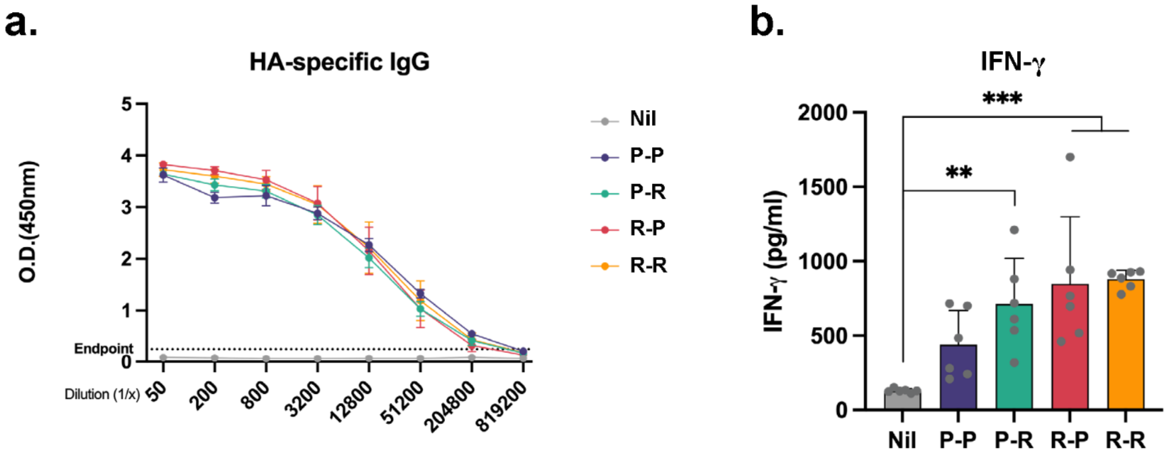

Supplementary Fig. 1. Humoral and cellular immune responses induced by homologous or heterologous priming-boosting. **a** Sera tested at different dilutions to detect IgG1 and IgG2c against HA (n = 6). **b** Measurement of IFN-γ levels in the splenocyte culture supernatant by indirect IFN-γ enzyme-linked immunosorbent assay (ELISA). Nil, virus-inoculated control; P-P, homologous protein-HA immunized; P-R, mRNA-HA boosting; R-P, mRNA-HA priming/protein-HA boosting; R-R, homologous mRNA-HA-immunized. Data are represented as the mean ± standard deviation (SD). Statistical significance was analyzed using one-way analysis of variance (ANOVA). The significance of differences between groups are indicated on the bars; ***P* < 0.01, ****P* < 0.005.

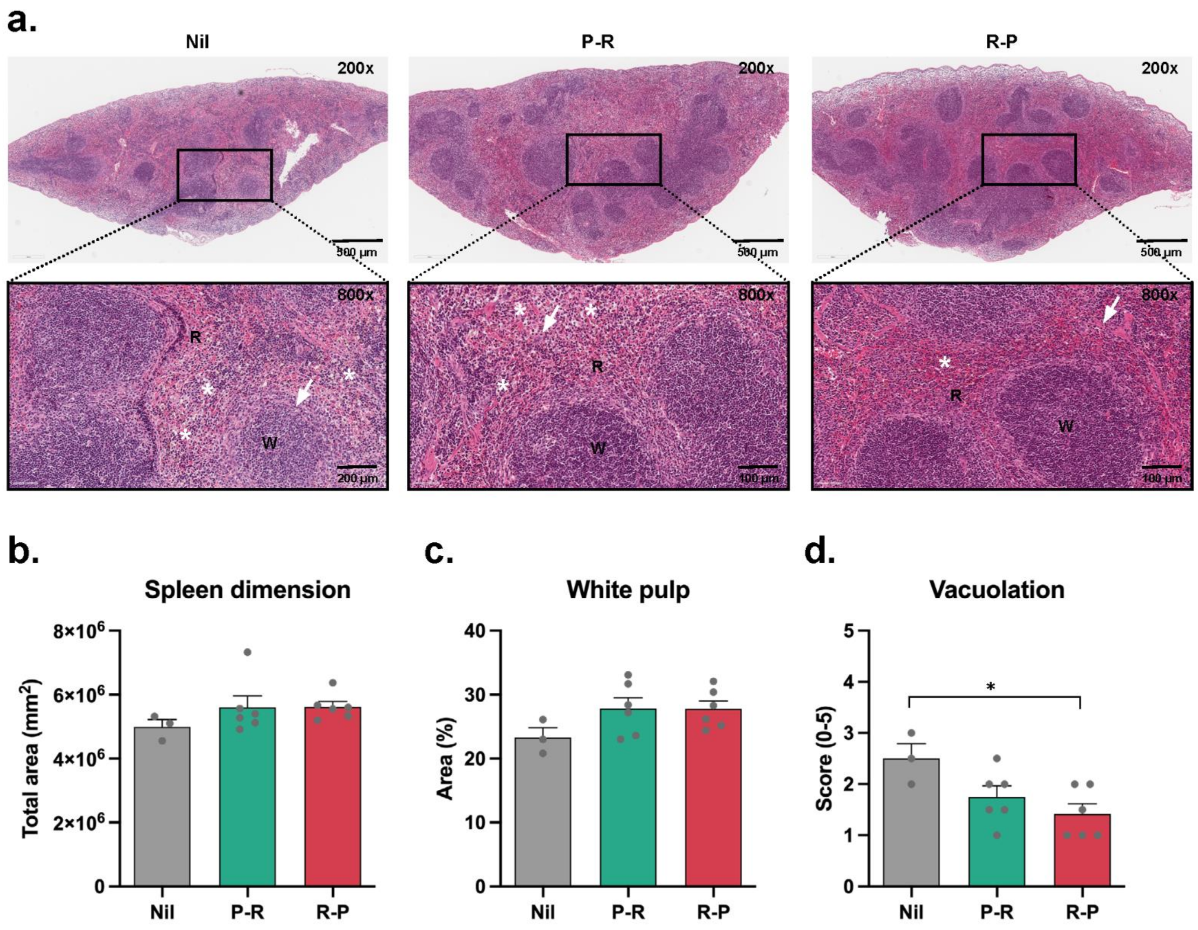

Supplementary Fig. 2. Histological analysis of the spleen of each immunized group after viral challenge. a Representative hematoxylin and eosin-stained images of the spleen of mice infected with influenza virus and sacrificed after 1 week. W, white pulp region; R, red pulp region; Arrows, megakaryocytes; Asterisks, vacuolation. **b** Total spleen dimension, **c** percentage of white pulp, and **d** vacuolation score. Nil, virus-inoculated control; P-R, mRNA-HA boosting; R-P, mRNA-HA priming/protein-HA boosting. Data are represented as the mean ± SD. Statistical significance was analyzed using one-way ANOVA. The significance of differences between groups are indicated on the bars; *P < 0.05.

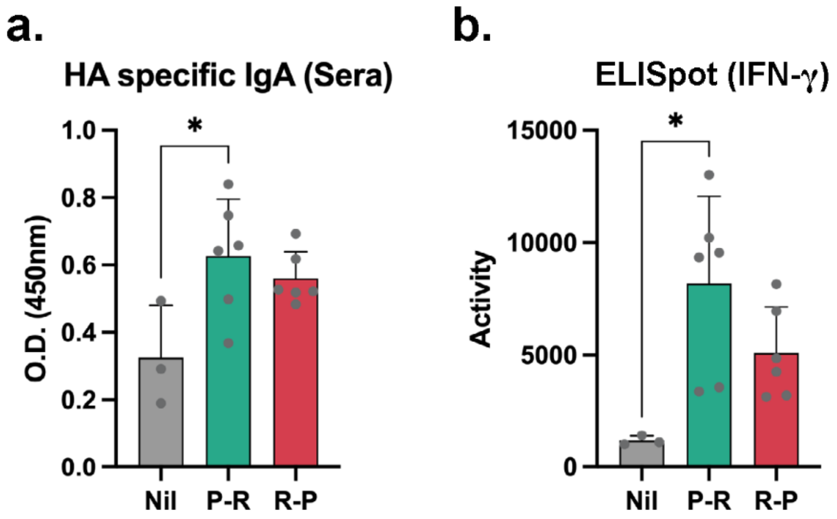

Supplementary Fig. 3. IgA levels and IFN-γ cytokine activity after viral challenge. **a** HA-specific IgA levels in the serum measured using indirect ELISA. **b** ELISpot assay showing IFN-γ-producing activity of mouse splenocytes stimulated with HA peptide pool. Nil, virus-inoculated control; P-R, mRNA-HA boosting; R-P, mRNA-HA priming/protein-HA boosting. Data are represented as the mean ± SD. Statistical significance was analyzed using one-way ANOVA. The significance of differences between groups are indicated on the bars; *$P < 0.05$.

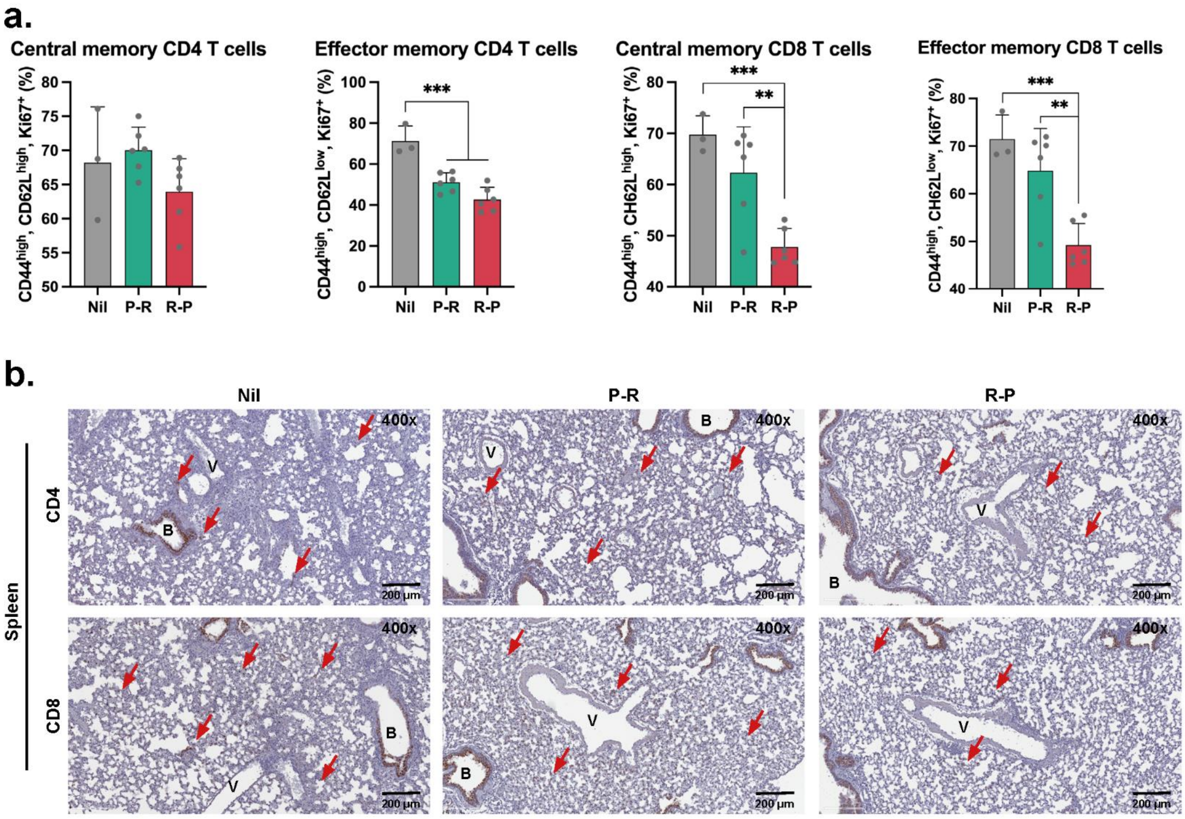

Supplementary Fig. 4. Analysis of T-cells after heterologous vaccination. BALB/c mice were intramuscularly primed and boosted with lipid nanoparticle-formulated mRNA-HA (5 μg) or AddaVax™-formulated HA protein (1 μg) at 2-week intervals. The mice were challenged with influenza PR8 virus 2 weeks after the boosting and sacrificed 1 week after the challenge. **a** Ki-67^{+} proliferating central and effector memory T-cells of CD4^{+} and CD8^{+} indicated by the activation markers. Data are represented as the mean ± SD. Statistical significance was analyzed using one-way ANOVA. The significance of differences between groups are indicated on the bars; $^{**}P < 0.01$, $^{***}P < 0.005$. **b** Immunohistochemical images of mouse lungs. Red arrows indicate CD4^{+} or CD8^{+} T-cells. B, bronchus or bronchi; V, blood vessels; Nil, virus-inoculated control; P-R, Protein-HA priming/mRNA-HA boosting; R-P, mRNA-HA priming/protein-HA boosting.

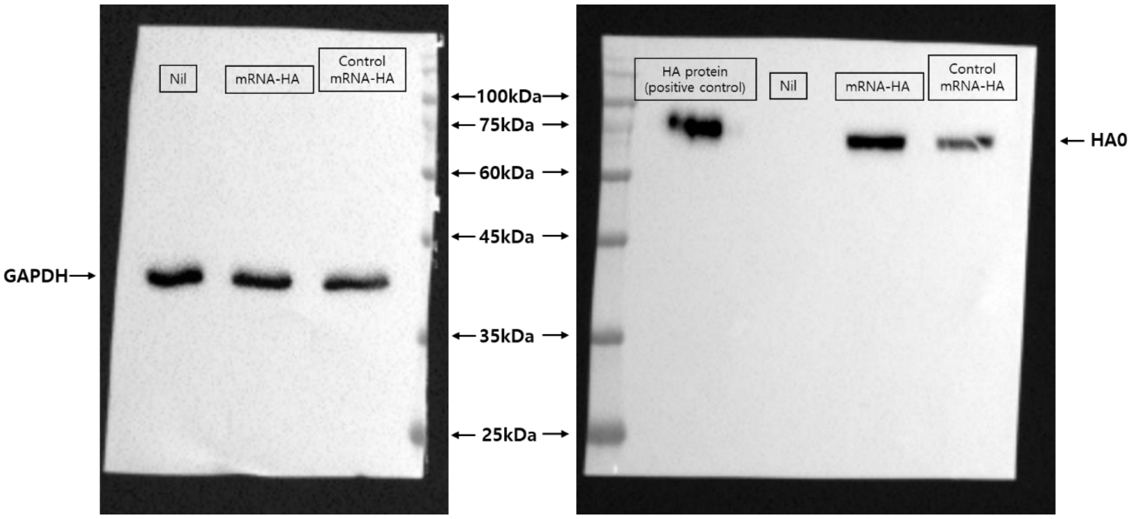

Supplementary Fig. 5. Expression of Hemagglutinin (HA) protein in mRNA-HA transfected Vero cells. Membrane image of a western blot to detect hemagglutinin (HA) proteins expressed in mRNA-HA transfected Vero cells. Left to right: Nil (untransfected sample), mRNA-HA used in the experiment, and control mRNA-HA.

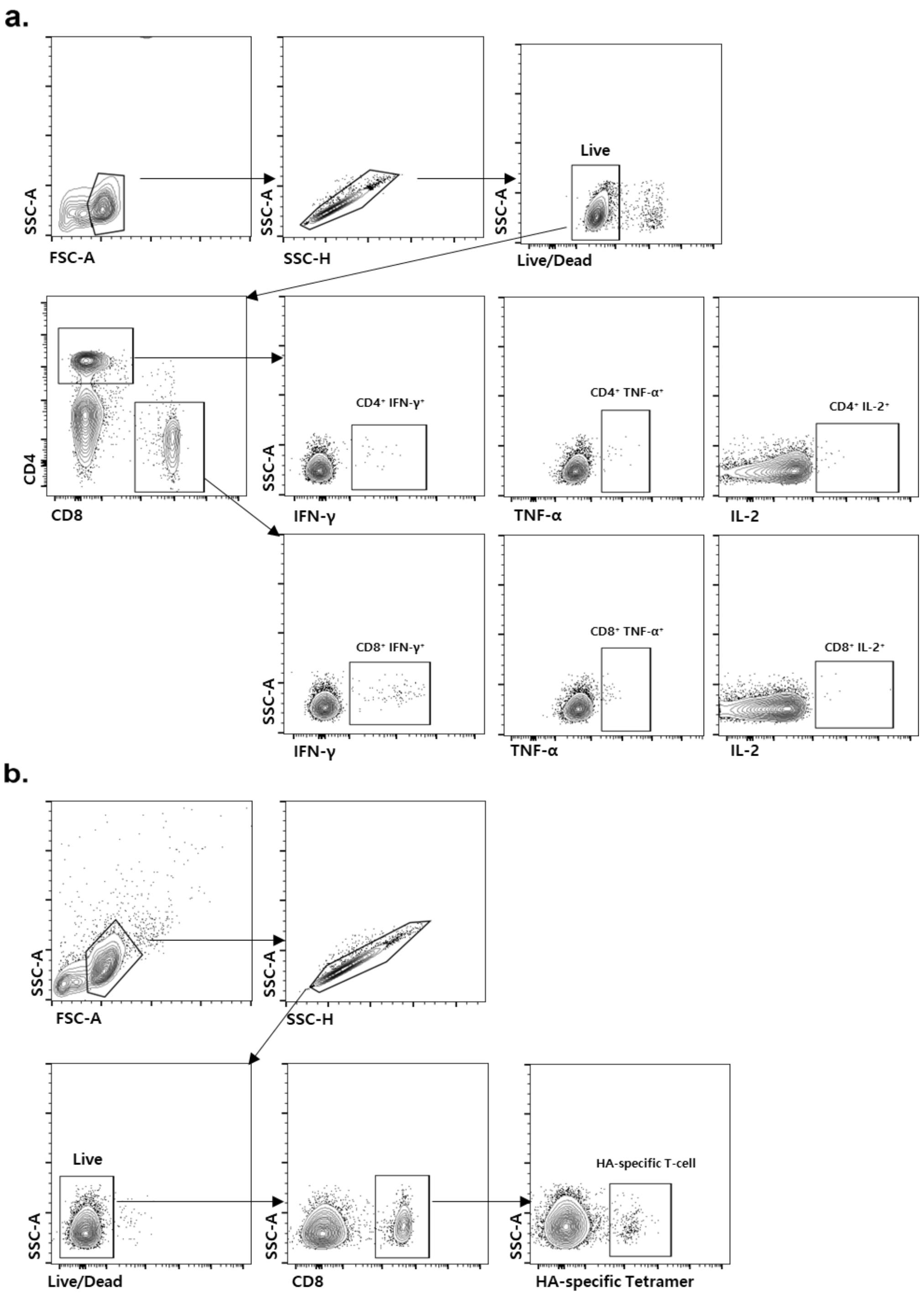

Supplementary Fig. 6. Gating strategies used for the T cell analysis. a Gating strategy to sort cytokine expression T-cells from immunized mice splenocytes. **b** Gating strategy to sort HA-specific T-cells from challenged mice splenocytes.

Supplemantary Table

Supplementary Table 1. mRNA-HA characteristics, including size, zeta potential, polydispersity index (PDI), and encapsulation efficiency.

	**LNP**
**Size (nm)**	97.0 ± 0.3
**PDI**	0.14
**Zeta potential (mV)**	27.6 ± 0.8
**Encapsulation efficiency**	87 %

LNP, lipid nanoparticle.
